# Wnt5a Promotes Axon Elongation in Coordination with the Wnt–Planar Cell Polarity Pathway

**DOI:** 10.3390/cells13151268

**Published:** 2024-07-28

**Authors:** Samar Ahmad, Liliana Attisano

**Affiliations:** Department of Biochemistry, Donnelly Centre, University of Toronto, Toronto, ON M5S 3E1, Canada; samar.ahmad@mail.utoronto.ca

**Keywords:** neuronal polarity, axon elongation, Wnt5a, planar cell polarity, extracellular vesicles

## Abstract

The establishment of neuronal polarity, involving axon specification and outgrowth, is critical to achieve the proper morphology of neurons, which is important for neuronal connectivity and cognitive functions. Extracellular factors, such as Wnts, modulate diverse aspects of neuronal morphology. In particular, non-canonical Wnt5a exhibits differential effects on neurite outgrowth depending upon the context. Thus, the role of Wnt5a in axon outgrowth and neuronal polarization is not completely understood. In this study, we demonstrate that Wnt5a, but not Wnt3a, promotes axon outgrowth in dissociated mouse embryonic cortical neurons and does so in coordination with the core PCP components, Prickle and Vangl. Unexpectedly, exogenous Wnt5a-induced axon outgrowth was dependent on endogenous, neuronal Wnts, as the chemical inhibition of Porcupine using the IWP2- and siRNA-mediated knockdown of either Porcupine or Wntless inhibited Wnt5a-induced elongation. Importantly, delayed treatment with IWP2 did not block Wnt5a-induced elongation, suggesting that endogenous Wnts and Wnt5a act during specific timeframes of neuronal polarization. Wnt5a in fibroblast-conditioned media can associate with small extracellular vesicles (sEVs), and we also show that these Wnt5a-containing sEVs are primarily responsible for inducing axon elongation.

## 1. Introduction

Neurons undergo distinct morphological changes during development. From round postmitotic cells, the neurons become polarized by acquiring a morphology comprised of a single axon and one or multiple dendrites [1]. The acquisition of this polarized neuronal morphology is critical for the structural and functional integrity of the CNS [2]. Isolated neurons from embryos or newborn rodents can polarize spontaneously upon culturing in vitro, and this model system has thus been widely used to study polarity events, including axon outgrowth [3,4]. Several extracellular factors, including Wnt family proteins, have been shown to modulate different aspects of neuronal polarization [2].

Wnts are the lipid-modified glycoproteins that signal through the Frizzled (Fzd) and non-Fzd receptors to activate either β-catenin-dependent canonical or β-catenin-independent non-canonical pathways [5,6]. In the nervous system, Wnts modulate diverse developmental processes, including axon guidance and outgrowth, dendritic morphology, and synapse formation [7,8,9]. Non-canonical Wnt5a can activate the Wnt–Planar Cell Polarity (PCP), Wnt–Ca^2+^, and other non-canonical pathways to promote axon formation and outgrowth. For instance, Wnt5a has been reported to promote axon outgrowth in commissural neurons through the PCP pathway, by activating aPKC through Dvl1 in hippocampal neurons or via the Wnt–Ca^2+^ pathway in postnatal (P2) and embryonic cortical neurons [10,11,12,13,14,15]. Wnt5a can also promote neurogenesis either by activating Rac1, a component of the Wnt–PCP pathway axis, in dopaminergic neurons [16] or through calcium/calmodulin-dependent kinase II (CaMKII), a Wnt–Ca^2+^ pathway component, in the adult hippocampus [17]. In addition, Wnt5a modulates dendritic spine formation through the Wnt–Ca^2+^ pathway by increasing the intracellular Ca^2+^. Wnt5a can signal through Fzd receptors via a non-canonical signaling axis. For instance, Wnt5a promotes dendritic branching through Fzd4 and increases the density of dendritic spines by activating CaMKII and c-Jun N-terminal kinase (JNK) via Fzd9 [18,19]. In addition to Fzds, Wnt5a can also signal through co-receptors, such as Ryk and Ror, either alone or in complex with Fzds [20,21]. For instance, Wnt5a acts as a repulsive guidance cue by modulating CaMKII through Ryk in the corpus callosum and the corticospinal tract, or by increasing Ca^2+^ activity through both Ryk and Fzd in postnatal cortical neurons [14,15,22,23]. Ryk has also been shown to be required for Wnt5a-induced axon outgrowth in postnatal cortical neurons, but interestingly, in a sub-population of cortical neurons, Wnt5a, acting through Ryk, inhibited axon outgrowth [14,24]. Thus, Wnt5a engages diverse receptors and intracellular mediators to control a diverse array of neuronal functions. Therefore, considerable uncertainty remains regarding the functions of Wnt5a. Being hydrophobic in nature, Wnts can be secreted in different ways, including in association with EVs. Wnt-conditioned media isolated from cells is a regularly used source of Wnt and contains EVs; however, it remains unanswered whether the Wnt5a-containing EVs contribute to neuronal morphology.

Here, we investigated the role of non-canonical Wnt5a in axon outgrowth and neuronal polarization in mouse embryonic cortical neurons. We found that non-canonical Wnt5a, but not canonical Wnt3a, promotes axon elongation. Axon outgrowth induced by exogenous Wnt5a was dependent on neuronal Wnts, as blocking the secretion of endogenous Wnts, either by chemically inhibiting Porcupine, an acyltransferase required for Wnt palmitoylation and secretion, or by the siRNA-mediated knockdown of Porcupine or Wntless, a Wnt-carrier protein required for the secretion of Wnts, resulted in the loss of the Wnt5a-induced elongation. Importantly, delayed treatment with the Porcupine inhibitor IWP2 did not prevent the Wnt5a-induced increase in the length of the axon. This suggests that the prior activity of endogenous Wnts in the time window when the axon specification typically occurs is required before Wnt5a can promote the elongation of the nascent axon. Thus, endogenous Wnts and exogenous Wnt5a each act during distinct time windows during neuronal polarization. The Wnt5a-induced effects required the activity of the core PCP components, including Prickle (Pk) 1/2 and Vangl1/2. Moreover, we confirmed previous reports [25,26] that Wnt5a in cell culture supernatants can associate with small extracellular vesicles (sEVs) and showed that these Wnt5a-containing sEVs substantially contributed to the axon elongation effects.

## 2. Materials and Methods

### 2.1. Animal Experiments

CD1 timed-pregnant mice were purchased from Charles River Laboratories and housed on a 12 h:12 h light:dark cycle with food and water provided ad libitum. All the experimental procedures were conducted in accordance with the guidelines of the University Animal Care Committee (UACC) of the University of Toronto, Ontario, Canada.

### 2.2. Cortical Neuron Isolation, Culturing, and Transfection

Primary cortical neurons were isolated from embryonic day (E) 15.5–16.5 CD1 mouse embryos according to a recently published protocol [27]. The dissociated neurons were seeded at a density of 20,000 cells/well in 8-well chamber slides (Ibidi, Gräfelfing, Germany, 80826) for the regular experiments or 125,000 cells/well for the transfection experiments in 4-well chamber slides (Lab-Tek II, Waltham, MA, USA, 155382) coated with poly-L-lysine (20 μg/mL, Sigma, Saint Louis, MI, USA) and laminin (2 μg/mL, Corning, New York, NY, USA). Neurons were grown at 37 °C and 5% CO_2_ in a serum-free neurobasal medium (Gibco, Waltham, MA, USA) supplemented with 2% B-27 (Gibco), 0.5% N-2 (Gibco), 2 mM GlutaMAX^TM^ (Gibco), and 0.5% penicillin/streptomycin (Gibco). The neurons were treated with the conditioned media (CM) or recombinant Wnt5a (R&D Systems, Minneapolis, MN, USA, 645-WN-010/CF) 4 h after plating. In the transfection experiments, the media was replaced with a fresh one 4 h after seeding and transfection.

For the transfection experiments, dissociated neurons were electroporated with siRNA using the Amaxa nucleofector system (program-005, Lonza, Basel, Switzerland) and the mouse neuron nucleofector^TM^ kit (VPG-1001, Lonza) according to the manufacturer’s specifications. The neurons (4–5 × 10^6^) were electroporated with 2 μg of enhanced green fluorescent protein (eGFP) as a transfection reporter and a mix of 2 μM siGENOME SMARTpool siRNAs (Dharmacon). The Dharmacon siRNAs are listed in Table 1.

### 2.3. Mammalian Cell Culture

Mouse L cells stably expressing Wnt3a (L-Wnt3a) and Wnt5a (L-Wnt5a) were previously generated from control L cells [28]. The MDA-MB-231 cells were purchased from ATCC. The L cells were grown in DMEM supplemented with 10% FBS. The L-Wnt3a and L-Wnt5a cells were grown in DMEM supplemented with 10% FBS and 0.4 mg/mL G418 (Thermo Fisher Scientific, Waltham, MA, USA, 10131035). The MDA-MB-231 cells were grown in a RPMI medium containing 5% FBS. All the cell lines were tested for mycoplasma contamination using a MycoAlert^TM^ Plus reagent (Lonza, LT07-703).

### 2.4. Preparation of the Conditioned Media

L cells, L-Wnt3a, and L-Wnt5a cells (0.5 × 10^6^) were cultured in a 100 mm dish and grown to confluency in their respective growth media as described above. Cells were washed with DMEM and incubated with DMEM without serum. The conditioned media was collected after 3 days, centrifuged at 300× *g* to remove cells, and stored at 4 °C. For the treatment of the neurons, the conditioned media was supplemented with 2% B-27, 0.5% N-2, 2 mM GlutaMAX^TM^, and 0.5% penicillin/streptomycin.

### 2.5. Isolation of the sEVs Using Differential Centrifugation

The small EVs were purified from 50 mL of the conditioned media using differential centrifugation. Briefly, the conditioned media was centrifuged at 300× *g* for 5 min and 2000× *g* for 20 min to remove the cells and large debris, respectively. The supernatants were centrifuged at 10,000× *g*, and the resulting supernatants were filtered through a 0.2 μm filter (Thermo Fisher Scientific). Subsequently, ultracentrifugation was performed at 100,000× *g* for 2 h using a type 70 Ti rotor (Beckman Coulter, Brea, CA, USA). The pellets were resuspended in 500 μL of sterile phosphate-buffered saline (PBS), diluted with 7.5 mL of PBS and then transferred to 16 × 76 mm polycarbonate ultracentrifuge tubes (Beckman Coulter, 355603). The tubes were centrifuged at 100,000× *g* for 2 h using a type 70.1 Ti rotor (Beckman Coulter). The pellets were resuspended in 250 μL of sterile PBS, aliquoted, and stored at −80 °C.

### 2.6. Isolation of the sEVs Using Density Gradient Purification

The small EV pellets (100,000× *g*) were purified using a discontinuous iodixanol density gradient that was prepared using a 60% stock solution of iodixanol (Optiprep^TM^, Sigma, D1556). The sEV pellets, resuspended in 500 μL of PBS, were overlaid on top of the discontinuous gradient (40%, 20%, 10%, and 5%) and centrifuged at 100,000× *g* for 16 h using a SW 40 Ti swinging bucket rotor (Beckman Coulter). Twelve individual fractions of 1 mL each were collected manually from the top, diluted with 7 mL of PBS, and transferred to polycarbonate ultracentrifuge tubes (Beckman Coulter, 355603). The tubes were centrifuged at 100,000× *g* for 2 h using a type 70.1 Ti rotor (Beckman Coulter), and the resulting pellets were resuspended in 150 μL of sterile PBS. The resuspended pellets were stored at −80 °C.

### 2.7. Nanoparticle Tracking Analysis

The size and number of particles of EVs were measured using the ZetaView^®^ PMX 110 device (Particle MetrixGmbH, Inning am Ammersee, Germany). The measurements were taken at all 11 different positions with the video quality set to medium, the camera sensitivity set to 90, and the shutter speed set to 45 using the ZetaView software (version 8.03.08). The analysis was performed using ZetaView analysis software (version 8.02.31) with the following post-acquisition parameters: minimum size 10, maximum size 1000, and minimum brightness 20.

### 2.8. Electron Microscopy

The small EVs were visualized using a Talos L120C transmission electron microscope (TEM) (Thermo Scientific) in the Microscopy Imaging Laboratory, University of Toronto, Canada. TEM grids (Quantifoil R2,2 300 mesh, EMS) were charged for 30 sec with a Pelco EasiGlow glow discharge cleaning system (Ted Pella, Redding, CA, USA) and the EV samples were frozen on the grids. The samples (4 μL) were deposited onto the grid and the grid was blotted and plunge frozen using a Vitrobot IV (FEI). Images were acquired at 28,000× and 57,000× magnifications at 120 kV. Images were collected at a defocus of −4 to −1.5 microns using the TEM Image and Analysis (TIA) software (version 5.0 SP4, Thermo Fisher Scientific).

### 2.9. Immunofluorescence Microscopy

Neurons were fixed in 4% paraformaldehyde for 30 min. The cells were permeabilized with 0.2% Triton/PBS for 5 min and blocked at room temperature for 1 h with 3% bovine serum albumin (BSA)/PBS. After blocking, the cells were incubated with a primary antibody overnight at 4 °C, followed by incubation with a secondary antibody for 2 h at room temperature. After washing three times, the cells were stored in PBS or an Ibidi mounting medium prior to imaging. For microscopy, the images were acquired using the 40X Plan-NEOFLUAR objective of the Zeiss Axiovert 200 M epifluorescence microscope. Confocal images were acquired using the Zeiss AXIO Observer Z1 microscope equipped with Zen Blue software (version 2, Zeiss, Oberkochen, Germany), a spinning disc confocal scanner (CSU-X1, Yokogawa, Tokyo, Japan), a CCD camera (Axiocam 506 mono), and a Plan-Apochromat 63x/1.4 Oil DIC M27 objective. The antibodies used for immunofluorescence are listed in Table 2.

### 2.10. Immunoblotting

The cells were lysed in lysis buffer (50 mM Tris-HCl, 150 mM NaCl, 1 mM EDTA, 0.5% Triton X-100, 1 mM DTT containing protease and phosphatase inhibitors) [30]. Proteins from the cell lysates (1–20 μg) and sEVs (1–10 μg) were separated on an SDS-PAGE gel and analyzed using western blotting using standard protocols. The antibodies for immunoblotting are listed in Table 2.

### 2.11. RT and qPCR

The total RNA was extracted from the regular neuronal cultures or FACS sorting of GFP-positive neurons using the Norgen Single-Cell RNA Purification Kit (Norgen, Thorold, ON, Canada, 51800), and the cDNA was synthesized using 35–500 ng of purified RNA using oligo-dT primers and M-MLV Reverse Transcriptase (Invitrogen, Waltham, MA, USA, 28025-013). Real-time qPCR was performed using the SYBR^TM^ Green PCR Master Mix (Applied Biosciences, Salt Lake City, UT, USA, 4309155) on the QuantStudio 6 Flex System (Applied Biosciences). The relative gene expression was quantified using the ΔΔCt method and normalized to glyceraldehyde-3-phosphate dehydrogenase (GAPDH). The primers used are listed in Table 3.

### 2.12. Quantification

For the neuronal morphology experiments, a minimum of 30 neurons per condition were quantified from each biological replicate. All the neurites, including the longest neurite (i.e., the prospective axon), the dendrites (all other neurites excluding the longest neurite), and the number of neurites, were quantified using the Volocity software (version 6.1.1, Quorum Technologies Inc., Puslinch, ON, Canada). In the MAP2 and Tau-1 experiments, the neuronal stages were defined based on a published criterion [31]. The neurons were considered in stage 1 (unpolarized) if without neurites or displaying neurites shorter than 10 μm. The neurons displaying neurites longer than 10 μm but without medial-to-distal Tau-1 accumulation were considered in stage 2 (unpolarized). A neurite longer than 10 μm but with a medial-to-distal accumulation of Tau-1, an axonal marker, was considered an axon. The neurons displaying single or multiple axons were considered in stage 3 (polarized).

### 2.13. Statistical Analyses

The data for the axon length and the longest neurite length were plotted as the average of the median ± SEM from 90–120 neurons per condition from three independent experiments. The data were analyzed statistically using an unpaired t-test for comparing two groups or a one-way ANOVA with Dunnett’s post-test for multiple comparisons to a single control group, or a two-way ANOVA with Tukey’s post-test for multiple comparisons to control and between the samples, as indicated in the figure legends. All the statistics were performed in GraphPad PRISM 9 (GraphPad software Inc., La Jolla, CA, USA). Statistical significance: * *p* < 0.05, ** *p* < 0.01, *** *p* < 0.001.

## 3. Results

### 3.1. Wnt5a Promotes Axon Outgrowth

**Figure 1:**
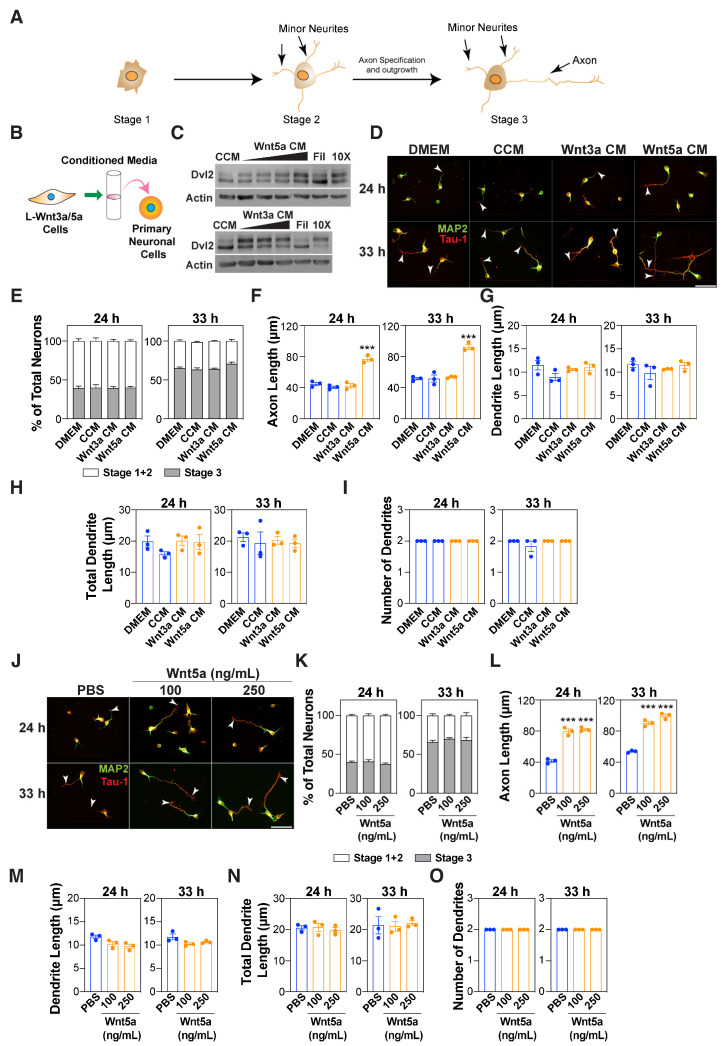
Wnt5a promotes axon outgrowth. (**A**) Stages of neuronal polarity. (**B**) A schematic of the experimental setup. Dissociated E15.5–16.5 mouse cortical neurons were treated with conditioned media (CM) isolated from control L cells and L cells stably expressing Wnt3a or Wnt5a. (**C**) Wnt3a- and Wnt5a-CM promote Dvl2 phosphorylation. MDA-MB-231 cells were treated with control-conditioned media (CCM), different dilutions of Wnt3a- or Wnt5a-CM, 10X concentrated (10X) CM, or the filtrate (Fil) for 4 h. Lysates were immunoblotted for Dvl2 and actin. (**D**–**O**) Cortical neurons were treated with Wnt-CM (**D**–**I**) or commercially available recombinant Wnt5a (100 and 250 ng/mL) (**J**–**O**), 4 h after plating. Neurons were fixed at 24 and 33 h, and the neuronal morphology was examined in neurons immunostained for MAP2 (dendrites, green) and Tau-1 (axons, red). Representative images are shown. Arrowheads mark the axon. Scale bar, 50 μm (**D**,**J**). (**E**–**I**,**K**–**O**) The average percentage of neurons in stages 1–3 (E, K), the length of the Tau-1-positive axon (**F**,**L**), the MAP2-positive individual dendrite lengths (**G**,**M**), the total dendrite lengths (**H**,**N**), and the total number of dendrites (**I**,**O**) were quantified. Values are plotted either as mean ± SEM (**E**,**K**) or an average of the median ± SEM from three independent experiments (**F**–**I**,**L**–**O**). In neuronal quantifications, each dot represents the median from at least 13 (for 24 h in panel F,L) or 30 (for all others) neurons from one of the three independent experiments. In panels F and L, for the 24 h group, the median was determined from a minimum of 13 neurons per experiment due to a greater percentage of neurons lacking a Tau-1-positive axon. Statistical significance: *** *p* < 0.001 using a one-way ANOVA with Dunnett’s post-test (**F**–**I**,**L**–**O**), or a two-way ANOVA with Tukey’s post-test (**E**,**K**).

Wnts play an important role in modulating axon guidance and outgrowth [8,32]. Wnt5a has been reported to either enhance or inhibit axon outgrowth [14,24]; therefore, its role in neuronal polarization remains unclear. Thus, we sought to examine the role of Wnt5a on axon outgrowth and neuronal polarity (Figure 1A) in mouse embryonic cortical neurons.

For this, a conditioned media (CM) from L cells stably expressing either Wnt5a (L-Wnt5a) or for comparison Wnt3a (L-Wnt3a), a canonical Wnt ligand, was prepared (Figure 1B). Wnt ligands induce Dvl phosphorylation, thus we first verified that active ligands were produced. For this, MDA-MB-231 cells were treated with various dilutions of the Wnt5a- and Wnt3a-conditioned media (CM), and the Dvl phosphorylation, as indicated by the upshift of Dvl2, was monitored using immunoblotting. As compared with the control-conditioned media collected from the parental L cells, both Wnt5a- and Wnt3a-conditioned media induced a Dvl2 upshift in a concentration-dependent manner that was strongest with the 10X concentrated media (Figure 1C). Next, the effect of the Wnt5a- and Wnt3a-CM on neuronal morphology was examined using dissociated E15.5–16.5 primary mouse embryonic cortical neurons. The neurons were treated 4 h after plating with a control and Wnt5a- or Wnt3a-CM for either 20 or 29 h and were fixed and stained with a Tuj1 antibody that detects the neuron-specific β-III tubulin. The quantification of the neurite lengths revealed that the Wnt5a-CM, but not Wnt3a-CM, increased the length of the longest neurite (i.e., the prospective axon) from ~30 μm in the controls to ~60 μm at 24 h and from ~50 μm in the controls to ~90 μm at 33 h (Figure S1A,B). In contrast, the individual length (Figure S1C) and total length (Figure S1D) of the minor neurites, (ie all the neurites except the longest neurite), which correspond to the prospective dendrite, remained unchanged. The number of neurites (Figure S1E) also remained unchanged. To confirm that the Wnt5a effect is specific to axons, the neurons were stained with antibodies against Tau-1, an axon marker, and MAP2, a dendritic marker. Wnt3a and Wnt5a did not affect the frequency of neuronal polarization as the percentage of neurons in stage 3 (ie see Figure 1A) remained unaltered (Figure 1D,E). The quantification of the neurite lengths revealed that Wnt5a-CM, but not Wnt3a-CM, enhanced the length of the Tau-1 positive axon from ~40 μm in the controls to ~70 μm at 24 h and from ~50 μm in the controls to ~90 μm at 33 h (Figure 1F). In contrast, the individual and the total combined length of the MAP2-positive dendrites, as well as the number of dendrites, remained unaltered (Figure 1G–I). As the effect of the Wnt5a-CM could arise from the presence of factors other than Wnt5a, these observations were validated using a commercially available, purified Wnt5a ligand. The treatment of cortical neurons with varying concentrations of recombinant Wnt5a increased the length of the Tuj1-stained longest neurite (Figure S1F–J) and Tau-1 positive axon without affecting the polarity at both 24 and 33 h in a dose-dependent manner (Figure 1J–L). Similar to the effects of the Wnt5a-CM, both the individual and total combined length of all the MAP2-positive dendrites and the number of dendrites remained unchanged when a purified Wnt5a ligand was used (Figure 1M–O). Taken together, these observations suggest that the non-canonical Wnt5a promotes axon outgrowth without affecting neuronal polarity.

### 3.2. Autocrine Wnts are Required for Wnt5a-Induced Axon Outgrowth

**Figure 2:**
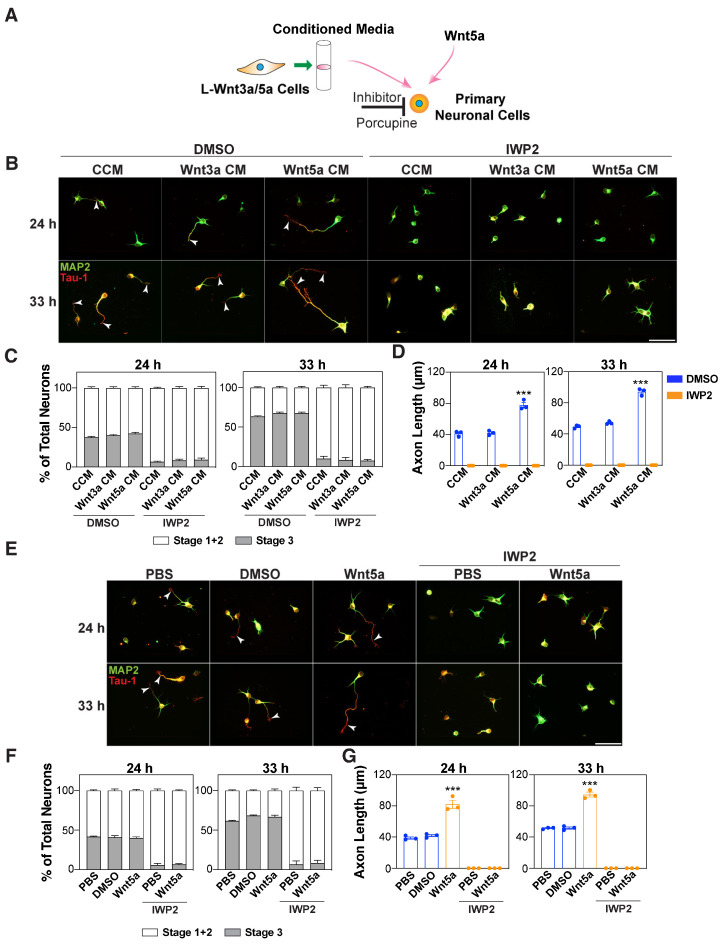
Autocrine Wnt signaling mediates Wnt5a-induced axon outgrowth. (**A**–**G**) Dissociated E15.5–16.5 mouse cortical neurons were treated with control-conditioned media (CCM) and Wnt3a- or Wnt5a-CM (**B**–**D**) or commercially available Wnt5a (100 ng/mL) (**E**–**G**) in the absence or presence of the Porcupine inhibitor, IWP2 (10 μM), 4 h after plating. Neurons were fixed at 24 and 33 h, and the neuronal morphology was examined in neurons immunostained for MAP2 (dendrites, green) and Tau-1 (axons, red). Representative images are shown. Arrowheads mark the axon. Scale bar, 50 μm (**B**,**E**). Average percentage of neurons in stages 1–3 (**C**,**F**) and the length of the Tau-1-positive axons (**D**,**G**) are plotted. Values are plotted either as mean ± SEM (**C**,**F**) or as an average of the median ± SEM from three independent experiments (**D**,**G**). In the neuronal quantifications, each dot represents the median from at least 13 (for 24 h in panel D,G) or 30 (for all others) neurons from one of the three independent experiments. In panels D and G, for the 24 h group, the median was determined from a minimum of 13 neurons per experiment due to a greater percentage of neurons lacking a Tau-1-positive axon. In panels D and G, no axon length data could be obtained for the IWP2-treated neurons as IWP2 blocked axon formation. Statistical significance: *** *p* < 0.001 using a one-way ANOVA with Dunnett’s post-test (**D**,**G**), or a two-way ANOVA with Tukey’s post-test (**C**,**F**).

Wnt ligands are secreted glycoproteins that are lipid-modified [33]. In Wnt-producing cells, the ER resident acyltransferase Porcupine (Porcn) is responsible for lipidating the Wnts, which is required for binding to Wntless (Wls), a Wnt carrier, for subsequent secretion [34,35]. Since the exogenous application of Wnt5a increased the length of the axon (Figure 1 and Figure S1), we next investigated the requirement for endogenous Wnts, by blocking Wnt secretion in neurons using the Porcupine inhibitor, IWP2 [36] (Figure 2A).

**Figure 3:**
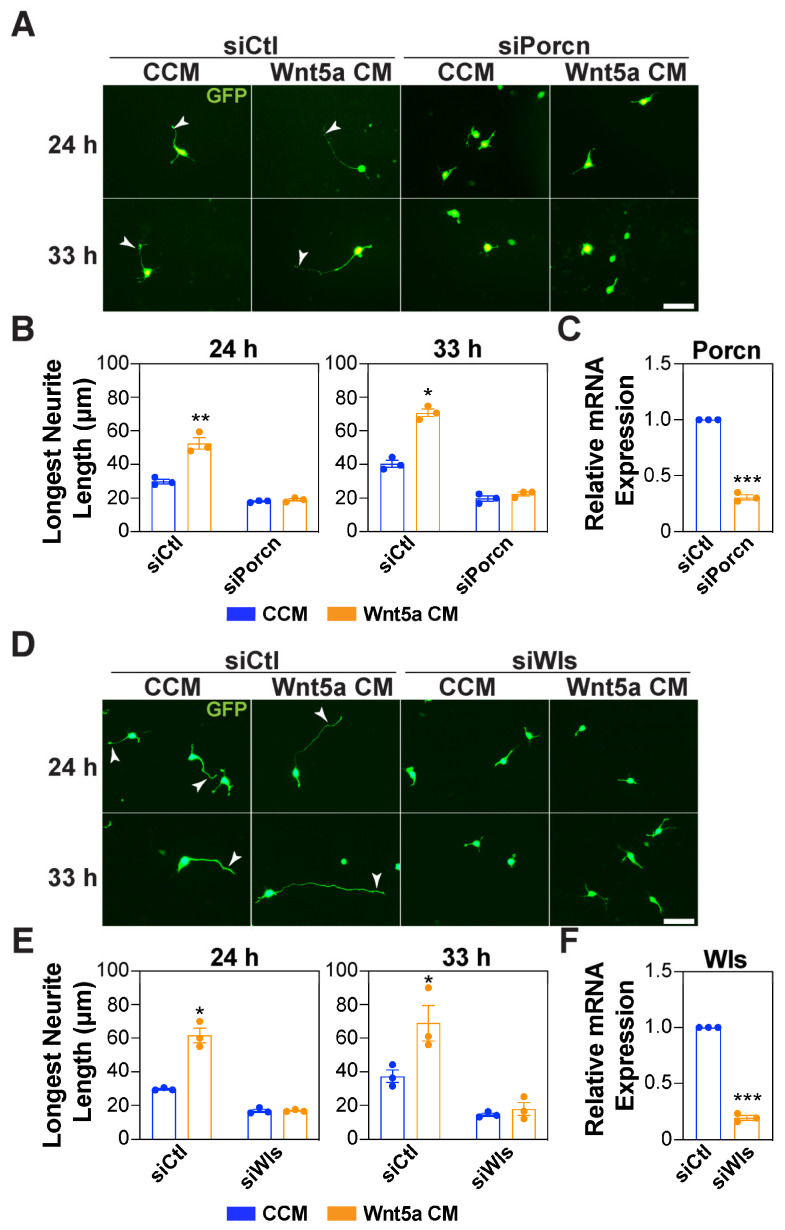
Autocrine Wnt signaling mediates Wnt5a-induced growth of the longest neurite. (**A**–**F**) Cortical neurons were electroporated with siRNAs against *Porcupine* (siPorcn) (**A**–**C**) or *Wntless* (siWls) (**D**–**F**) or siControl (siCtl) along with a GFP-expressing plasmid and then treated with CCM or Wnt5a-CM, 4 h after plating. Neurons were fixed at 24 and 33 h, and the neuronal morphology was examined in GFP-positive neurons. Representative images (**A**,**D**) and quantifications of the longest neurite (**B**,**E**) are shown. Arrowheads mark the longest neurite. Scale bar, 40 μm. (C,F) Knockdown efficiency for *Porcupine* (**C**) and *Wls* (**F**) was determined in GFP-positive neurons isolated using FACS. Relative mRNA expression was determined by qPCR. Values are plotted as mean ± SEM (**C**,**F**) or as an average of the median ± SEM from three independent experiments (**B**,**E**). In the neuronal quantifications, each dot represents the median from a minimum of 30 neurons from one of the three independent experiments (**B**,**E**). Statistical significance: * *p* < 0.05, ** *p* < 0.01, *** *p* < 0.001 using an unpaired t-test (**C**,**F**), or a two-way ANOVA with Tukey’s post-test (**B**,**E**).

The treatment of neurons with IWP2 inhibited the frequency of neuronal polarization in dissociated cortical neurons as indicated by the lack of a Tau-1-positive axon (Figure 2B,C). Moreover, concurrent treatment with Wnt5a-CM and IWP2 prevented the formation of a Tau-1-positive axon, and accordingly, Wnt5a-induced axon elongation was not observed (Figure 2C,D). Similarly, in the case of purified Wnt5a, the concurrent treatment of neurons with IWP2 inhibited the frequency of neuronal polarization and impaired the Wnt5a-mediated elongation effect (Figure 2E–G and Figure S2A,B). We also examined the effect of blocking the secretion of endogenous Wnts using siRNAs that target either *Porcupine* or *Wntless (Wls)*. Ablating the expression of *Porcupine* or *Wntless* using siRNAs similarly inhibited outgrowth of the longest neurite, a prospective axon, induced by Wnt5a-CM (Figure 3A–F). The knockdown efficiency for *Porcupine* and *Wntless* was confirmed using qPCR after FACS sorting for eGFP-positive neurons (Figure 3C,F). Thus, blocking the secretion of endogenous Wnts prevents the acquisition of a polarized neuronal morphology and impairs the Wnt5a-induced outgrowth of the longest neurite.

### 3.3. Wnt5a Promotes Axon Elongation in a Distinct Timeframe

**Figure 4:**
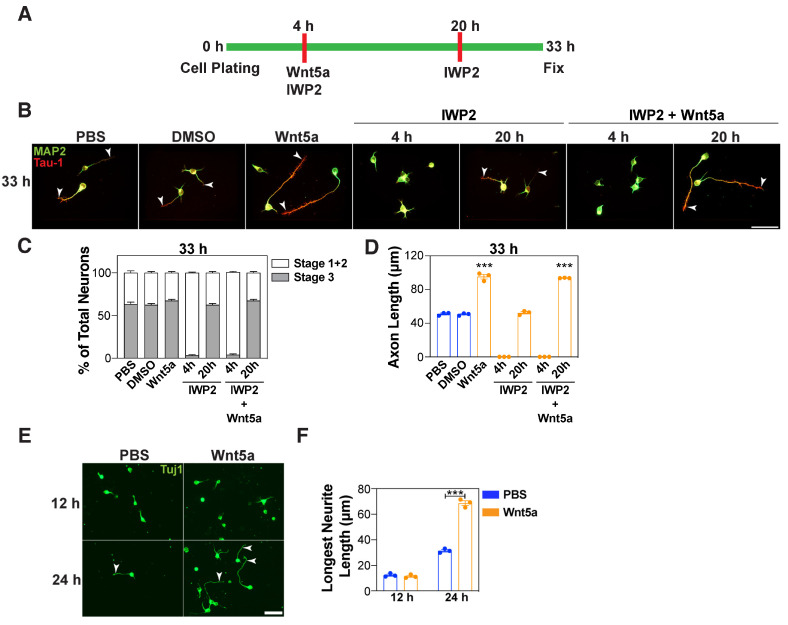
Wnt5a promotes axon elongation in a distinct timeframe. (**A**–**D**) Dissociated E15.5–16.5 mouse cortical neurons were treated with Wnt5a (100 ng/mL), 4 h after plating, in the absence or presence of IWP2 (10 μM), added 4 h or 20 h after plating. (**E**,**F**) Neurons were treated with Wnt5a (100 ng/mL), 4 h after plating. Neurons were fixed at 33 h (**A**–**D**) or 12 and 24 h (**E**,**F**) and the neuronal morphology was examined in Tau-1/MAP2-stained (**B**–**D**) or Tuj1-stained neurons (**E**,**F**). (**B**,**E**) Representative images are shown. Arrowheads mark the longest neurite. Scale bar, 40 μm (**E**) or 50 μm (**B**). Average percentage of neurons in stages 1–3 (**C**), the length of the Tau-1-positive axons (**D**), and the length of the longest neurite (**F**) were quantified. Values are plotted either as mean ± SEM (**C**) or as an average of the median ± SEM from three independent experiments (**D**,**F**). In the neuronal quantifications, each dot represents the median from a minimum of 30 neurons from one of the three independent experiments (**D**,**F**). In panel (**D**), no axon length data could be obtained for the IWP2-treated neurons at 4 h, as IWP2 blocked axon formation. Statistical significance: *** *p* < 0.001 using a one-way ANOVA with Dunnett’s post-test (**D**), or a two-way ANOVA with Tukey’s post-test (**C**,**F**).

To further examine how endogenous Wnt secretion is linked to Wnt5a-induced outgrowth of the prospective axon, we next investigated the effect of blocking Wnt secretion at different phases of neuronal polarization. For this, we compared the effect of adding IWP2 to the neuronal cultures both during and after Wnt5a treatment (Figure 4A). As observed above (Figure 2), the addition of IWP2 together with Wnt5a, 4 h after plating, inhibited the outgrowth of the longest neurite (Figure S2C,D) and prevented axon formation and impaired the Wnt5a-induced axon elongation at 33 h (Figure 4B–D). In contrast, the addition of IWP2 at 20 h after plating (i.e., 16 h after Wnt5a addition) did not affect neuronal polarization and resulted in an increase in the axon length at 33 h. This indicates that the secretion of neuronal Wnts is critical in the early phase, but not in the later phase of neuronal polarization. Moreover, exogenous Wnt5a appears to act in a distinct (later) timeframe, to promote axon elongation. Consistent with this, the analysis of the neurites at 12 h after plating, when the neurites are short, revealed that Wnt5a had no effect on neurite lengths (Figure 4E,F), which contrasts with the enhanced elongation observed at 24 and 33 h (Figure 1F,L, Figure 2D,G and Figure 3B,E). Taken together, these observations suggest that endogenous Wnts and exogenous Wnt5a each might act during specific time windows to control neuronal polarization and axon outgrowth.

### 3.4. Wnt5a-Induced Outgrowth of the Prospective Axon Requires Coordination with PCP Components

**Figure 5:**
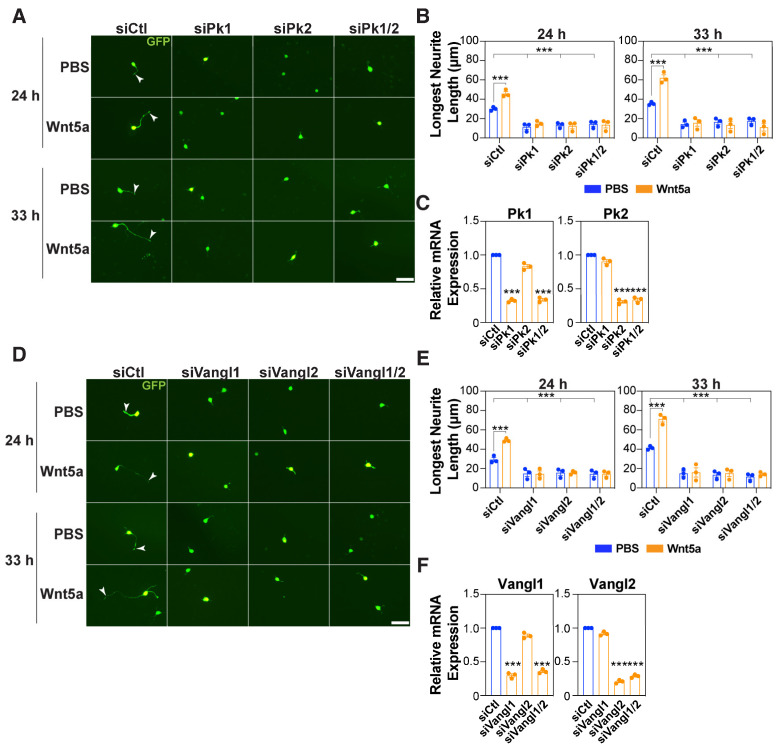
The activity of the PCP components, Prickle and Vangl, is required for Wnt5a-induced neurite outgrowth. (**A**–**F**) Dissociated E15.5–16.5 mouse cortical neurons were electroporated with siRNAs against *Pk1* (siPk1) and *Pk2* (siPk2) (**A**–**C**), or *Vangl1* (siVangl1) and *Vangl2* (siVangl2) (**D**–**F**) individually or in combination or with siControl (siCtl) along with a GFP-expressing plasmid and then treated with Wnt5a (100 ng/mL), 4 h after plating. Neurons were fixed at 24 and 33 h, and the neuronal morphology was examined in GFP-positive neurons. Representative images (**A**,**D**) and quantifications of the longest neurite (**B**,**E**) are shown. Arrowheads mark the longest neurite. Scale bar, 40 μm. (**C**,**F**) Knockdown efficiency for *Pk1*/*Pk2* (**C**) and *Vangl1/2* (**F**) was determined in GFP-positive neurons isolated using FACS. Relative mRNA expression was determined using qPCR. Values are plotted either as mean ± SEM (**C**,**F**) or as an average of the median ± SEM from three independent experiments (**B**,**E**). In the neuronal quantifications, each dot represents the median from a minimum of 30 neurons from one of the three independent experiments (**B**,**E**). Statistical significance: *** *p* < 0.001 using a one-way ANOVA with Dunnett’s post-test (**C**,**F**), or a two-way ANOVA with Tukey’s post-test (**B**,**E**).

Wnt5a can activate non-canonical Wnt signaling pathways, including the Wnt–PCP pathway. Therefore, we next sought to explore the signaling pathway underlying the Wnt5a-induced outgrowth of the cortical neurons. To study this, we used siRNA-mediated knockdown to investigate the contribution of the core PCP components, Pk and Vangl, which are known to modulate different aspects of neuronal morphology [11,37,38,39]. To test for the requirement for Pk and Vangl, neurons were co-transfected with siRNAs targeting *Pk1, Pk2, Vangl1,* or *Vangl2* along with eGFP to mark the transfected cells. The analysis of the eGFP-positive neurons in which the expression of *Pk1* and *Pk2* alone or together was abrogated revealed that the neurons were comprised solely of minor neurites (median length ~10 μm), none of which were elongated upon treatment with Wnt5a (Figure 5A,B). Similarly, the downregulation of *Vangl1* and *Vangl2* resulted in neurons containing only minor neurites (median length ~8 μm), and Wnt5a-mediated outgrowth was inhibited (Figure 5D,E). The knockdown efficiency for *Pk* and *Vangl* was confirmed using qPCR after FACS sorting for eGFP-positive neurons (Figure 5C,F). These observations indicate that Wnt5a requires coordination with the PCP components, Pk and Vangl, to promote the growth of the longest neurite.

### 3.5. Wnt5a-Containing sEVs Promote Axon Elongation

**Figure 6:**
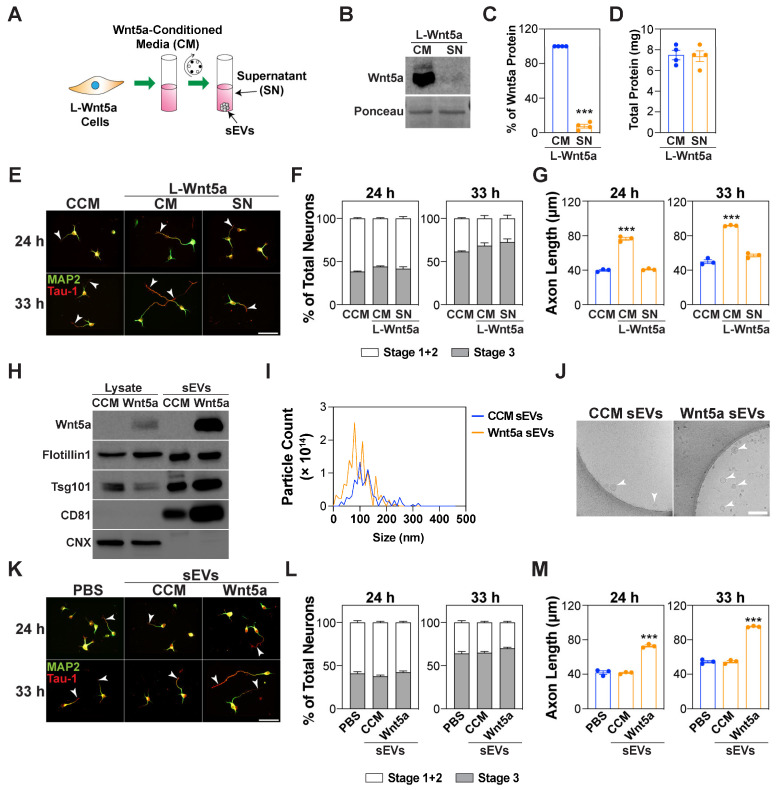
Wnt5a-containing sEVs promote axon outgrowth. (**A**) A schematic of the experimental setup. Small EVs (100,000× *g* pellet) were purified from Wnt5a-CM using differential centrifugation. (**B**–**D**) Wnt5a is predominantly present in the sEV pellet. Representative immunoblot of Wnt5a (**B**), respective quantification (**C**), and total protein (**D**) in 50 mL of the original Wnt5a-CM (CM) and the sEV-depleted supernatant (SN) obtained after ultracentrifugation at 100,000× *g* from four independent batches is shown. (**E**–**G**) sEV-depleted Wnt5a-CM cannot promote axon outgrowth. (**H**) Representative immunoblotting of the cell lysates and sEV pellets (100,000× *g*) for the EV markers CD81, Flotillin1, and TSG101 and the ER protein, calnexin (CNX). (**I**) A nanoparticle tracking analysis (NTA) of the differential centrifugation pellets. A representative plot indicating the particle size distribution from three independent purifications is shown. (**J**) Representative transmission electron microscopy (TEM) images of the sEV-containing pellets. Arrowheads indicate round vesicles. Scale bar, 200 nm. (**K**–**M**) Wnt5a-containing sEVs promote axon outgrowth. (**E**–**G**,**K**–**M**) Cortical neurons were treated with sEV-containing or sEV-depleted Wnt5a-CM (**E**–**G**), and sEVs from control-conditioned media (CCM) or Wnt5a-CM (all at 5 μg/mL) (**K**–**M**), 4 h after plating. Neurons were fixed at 24 and 33 h, and the neuronal morphology was examined in Tau-1-/MAP2-stained neurons. Representative images (**E**,**K**) and quantifications indicating the average percentage of neurons in stages 1–3 (**F**,**L**) and Tau-1-positive axon lengths (**G**,**M**) are shown. Arrowheads mark the axon. Scale bar, 50 μm (**E**,**K**). Values are plotted either as mean ± SEM (**C**,**D**,**F**,**L**) or as an average of the median ± SEM from three independent experiments (**G**,**M**). In neuronal quantifications, each dot represents the median from at least 13 (for 24 h in panel **G**,**M**) or 30 (for all others) neurons from one of the three independent experiments. In panels G and M, for the 24 h group, the median was determined from 13 neurons per experiment due to a greater percentage of neurons lacking a Tau-1-positive axon. Statistical significance: *** *p* < 0.001 using an unpaired *t*-test (**C**,**D**), a one-way ANOVA with Dunnett’s post-test (**G**,**M**), or a two-way ANOVA with Tukey’s post-test (**F**,**L**).

Previous studies have reported that active Wnt proteins can associate with exosomes, which is considered a mode of intercellular Wnt transport, and can thereby activate signaling pathways in recipient cells [25,26]. To investigate this in our system, we examined the association of Wnt5a with the EVs in cell culture supernatants from L cells stably expressing Wnt5a (L-Wnt5a). For this, we first isolated the sEVs from the Wnt5a-conditioned media using differential centrifugation (Figure 6A).

A western blot analysis of the Wnt5a-conditioned media before and after ultracentrifugation revealed that roughly 90% of the Wnt5a was present in the sEV pellet (Figure 6B,C). In contrast, the total protein amount remained unaltered in the sEV-containing and sEV-depleted media, indicating that the reduction in the Wnt5a level in sEV-depleted media was not due to a general decrease in protein amounts (Figure 6D). Accordingly, the treatment of neurons with the sEV-depleted media showed that the activity of the Wnt5a-CM in promoting axon elongation was lost upon sEV depletion (Figure 6E–G). Next, we characterized the sEV-containing pellet, which was purified using differential ultracentrifugation. The analysis of the sEV pellet, derived from L-Wnt5a cells, using immunoblotting showed the presence of Wnt5a along with EV markers including CD81, TSG101, and Flotillin1, and the absence of the endoplasmic reticulum (ER) marker Calnexin (CNX) (Figure 6H). As expected, Wnt5a was absent in the sEV pellet derived from the control L cells (Figure 6H). The analysis of the particle size using a nanoparticle tracking analysis (NTA) revealed that the majority of the EVs were within a size range of 30–150 nm (Figure 6I). Moreover, the ultrastructural analysis using transmission electron microscopy (TEM) showed intact round vesicles (Figure 6J), confirming the structural integrity of the sEVs. The treatment of cortical neurons with sEVs revealed that although the Wnt5a-containing sEVs had no statistically significant effect on the expression level of the PCP components, Pk and Vangl, the Wnt5a-containing sEVs, but not the control sEVs, promoted axon elongation without affecting neuronal polarization (Figure S3 and Figure 6K–M).

**Figure 7:**
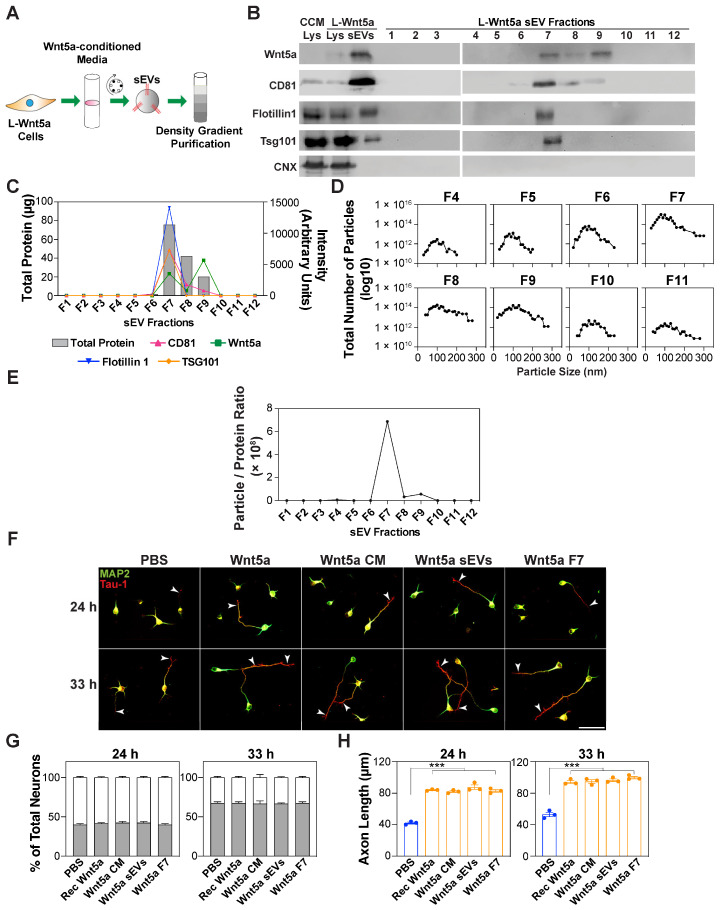
Wnt5a-containing sEVs isolated using density gradient purification promote axon outgrowth. (**A**) A schematic of the experimental setup. Small EV pellets (100,000× *g*) purified from the conditioned media (CM) of L-Wnt5a cells using differential centrifugation were subjected to iodixanol discontinuous density gradient purification. (**B**) Wnt5a is present in fractions containing sEVs. A small EV pellet (100,000× *g*) was loaded on top of a discontinuous gradient and ultracentrifuged for 16 h. Twelve fractions were collected from the top and analyzed using immunoblotting for Wnt5a, the ER protein calnexin (CNX), and EV markers, including CD81, Flotillin1, and TSG101. (**C**) The intensity of the bands from (**B**) is plotted against the amount of the protein. (**D**) Particle size distribution was determined using NTA. (**E**) Fraction 7 (F7) has the highest particle number/protein ratio. (**F**–**H**) F7 fraction promotes axon elongation. Cortical neurons were treated with recombinant Wnt5a (100 ng/mL), Wnt5a-CM, crude sEVs (5 μg/mL), or F7 fraction (5 μg/mL), 4 h after plating. Neurons were fixed at 24 and 33 h, and the neuronal morphology was examined in Tau-1-/MAP2-stained neurons. Representative images (**F**) and quantifications indicating the average percentage of neurons in stages 1–3 (**G**) and Tau-1-positive axon lengths (**H**) are shown. Arrowheads mark the axon. Scale bar, 50 μm (**F**). Values are plotted either as mean ± SEM (**G**) or an average of the median ± SEM from 3 independent experiments (**H**). In the neuronal quantifications, each dot represents the median from at least 13 (for 24 h in panel **H**) or 30 (for all others) neurons from one of the three independent experiments. In panel H, for the 24 h group, the median was determined from 13 neurons per experiment due to a greater percentage of neurons lacking a Tau-1-positive axon. Statistical significance: *** *p* < 0.001 using a one-way ANOVA with Dunnett’s post-test (**H**), or a two-way ANOVA with Tukey’s post-test (**G**).

Next, to rule out the role of protein aggregates and contaminating cell membranes, the crude sEV pellet obtained after ultracentrifugation was further purified using iodixanol density gradient centrifugation, which separates the co-sedimenting molecules based on their density (Figure 7A). The characterization of the floating fractions using immunoblotting revealed the presence of EV markers, including CD81, TSG101, and Flotillin1, predominantly in fraction 7, whereas the ER-resident protein, Calnexin, was absent (Figure 7B). Wnt5a was found in the crude ultracentrifugation pellet and fraction 7 of the subsequent density gradient purification, indicating that Wnt5a is associated with sEVs (Figure 7B,C). Wnt5a was also present in fractions 8 and 9 that lacked EV markers, and may reflect the Wnt5a associated with denser vesicles or with high-density lipoprotein particles. The analysis of the particle size showed that the number of particles peaked roughly at around 100 nm, and that fraction 7 had the highest number of particles (Figure 7D). In addition, Fraction 7 had the highest particle/protein ratio (Figure 7E), which is an indicator of the purity of the EVs. The treatment of the neurons with this pure Wnt5a-containing F7 fraction promoted axon elongation, without affecting neuronal polarization, to levels similar to those observed for the original Wnt5a-conditioned media and the crude EV pellet (Figure 7F–H). These observations indicate that the axon elongation induced by Wnt5a-CM is in part largely due to the Wnt5a associated with the sEVs.

**Figure 8:**
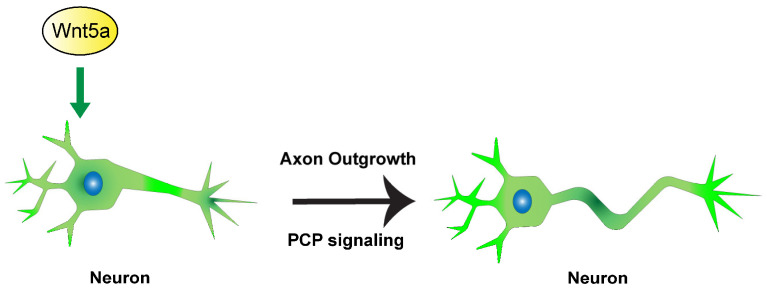
Wnt5a promotes axon elongation through Wnt–PCP signaling. Wnt5a promotes axon elongation without affecting neuronal polarization, and this Wnt5a-induced effect is lost upon the loss of expression of the PCP components, Pk and Vangl.

Taken together, our data indicate that Wnt5a promotes the elongation of the newly specified axon and this effect of Wnt5a requires the secretion of neuronal Wnts and the activity of PCP components during the early stages of neuronal polarization in embryonic cortical neurons (Figure 8).

## 4. Discussion

Wnt5a, a non-canonical ligand, exhibits varying roles in axon outgrowth and diverse signaling pathways can mediate the Wnt5a effects. In this study, we explored the role of Wnt5a in axon outgrowth in embryonic cortical neurons. We found that non-canonical Wnt5a, but not canonical Wnt3a, promotes axon outgrowth and does so through the prior activity of the core PCP components, Pk and Vangl. Moreover, we showed that while endogenous Wnts are essential during the early stages of neuronal polarization, endogenous Wnts are dispensable later, for Wnt5a-induced axon elongation. Thus, based on our results, we speculate that endogenous Wnts and Wnt5a might act during early and late time windows, respectively, to modulate neuronal polarization and axon outgrowth.

In this study, we found that Wnt5a promotes axon outgrowth but had no effect on neuronal polarization, the individual or total dendrite length, or the number of dendrites, which is consistent with several reports showing that Wnt5a promotes axon outgrowth in various types of neurons grown in vitro, including mouse postnatal cortical and rat embryonic cortical neurons [12,13,14,15]. Conversely, one report showed that Wnt5a inhibited axon outgrowth in a subpopulation of cortical neurons [24]. Although the in vivo validation of the axon outgrowth effects of Wnt5a remains to be explored, nevertheless, it appears that there is a complex role for Wnt5a in different neuronal populations. Our data indicate that exogenous Wnt3a does not appear to affect axon outgrowth in dissociated cortical neurons, which is contradictory to reports showing that Wnt3a promotes neurite outgrowth in DRG neurons and neuronal polarization in hippocampal neurons [40,41]. This suggests that Wnt3a displays cell type-specific effects in promoting axon outgrowth, and thus more studies are needed to address the differential role of Wnt3a.

Wnts are expressed throughout the CNS and play an important role in modulating processes that establish neuronal morphology and function [8,32,42,43]. Our data showed that neuronal Wnts are required for neuronal polarization in the early stages but are dispensable later, which is consistent with a previous report showing that early treatment with the porcupine inhibitor IWP2 inhibits neuronal polarization [41]. Moreover, we observed that Wnt5a-induced axon elongation was impaired in the presence of IWP2 during an early timeframe, which is typically associated with axon specification events, but not at later times that are generally associated with the elongation of the nascent axon. Accordingly, we observed that Wnt5a can increase the length of the longest neurite at later time points, but not at early times. This suggests that endogenous Wnts and exogenous Wnt5a each act in distinct time windows during the process of neuronal polarization.

Wnt5a is expressed in the brain cortex and has been shown to simultaneously promote axon outgrowth while acting as a repulsive axon guidance cue [14,15,23]. It was shown that Wnt5a signals through Ryk, a receptor tyrosine kinase, when acting as a repulsive cue, whereas Wnt5a signals through Ryk and Fzd to promote axon outgrowth [14,15]. In Drosophila, Wnts are not required to activate PCP signaling; however, growing evidence indicates that in mammals, Wnts do activate PCP signaling [44]. We found that the core PCP components, Pk and Vangl, are required for neurite outgrowth, and that Wnt5a-induced effects required the activity of the core PCP components. We speculate that the PCP components, Pk and Vangl, and Wnt5a might act during early and later timeframes, respectively, of neuronal development. Indeed, the PCP component Vangl2 is required for Wnt5a-induced axon elongation in commissural neurons [11]. In contrast, a study in hippocampal neurons showed that neurite outgrowth is increased in the absence of Vangl2 and that Vangl2 is not required for Wnt5a-induced axon outgrowth [45]. Thus, it is possible that PCP signaling components might not use a conserved signaling mechanism in different types of neurons and thus may respond differently to Wnt5a. This differential response has been observed in Fzd3-knockout mice, where different types of axons exhibit different defects in axonal growth and guidance [46]. Other reports have shown that Wnt5a activates the Wnt–Ca^2+^ pathway through Ryk in promoting axon outgrowth in rat embryonic and postnatal cortical neurons [12,13,14,15]. Moreover, under certain conditions, Wnt5a has also been shown to activate the canonical Wnt β-catenin signaling pathway, such as when Fzd4 is overexpressed [47,48]. Thus, we envision that Fzd and non-Fzd receptor availability could be a factor in determining whether the Wnt–PCP, Wnt–Ca^2+^, or other pathways are activated in response to Wnt5a.

Wnts are hydrophobic in nature, and thus various mechanisms have been proposed for the long-range transport of Wnts. One such mechanism is through extracellular vesicles, such as exosomes [49]. Our data show that Wnt5a can associate with sEVs, which is consistent with previous reports indicating that Wnts are secreted on exosomes [25,26]. We observed that roughly 90% of the Wnt5a present in the conditioned media is found in the sEV pellet. Further analysis of the sEV pellet showed that Wnt5a is indeed associated with sEVs. Thus, it is possible that the effects observed using Wnt5a-CM could be the result of Wnt5a associated with or found within sEVs. A previous report showed that roughly 40% of the total Wnt3a is present in the exosome pellet, and that exosomal Wnt can activate downstream signaling in the recipient cells [26]. Some Wnt5a has also been shown to be secreted on microvesicles, which are larger vesicles that directly bud from the cell membrane [50]. We also observed the presence of Wnt5a in denser sEV fractions, which could indicate Wnt5a associated with higher density sEVs or with lipoprotein particles. Wnts bound to EVs are relatively more stable than free Wnts, and thus may represent a means to increase the availability of the Wnt ligand [51,52]. We found that Wnt5a-containing sEVs are active in promoting axon elongation, similar to that of Wnt5a-CM, which is in agreement with a report indicating that Wnts associated with EVs are biologically active [26]. Additional activity assays showed that Wnt5a-containing sEVs did not alter the expression level of the PCP components, Pk and Vangl. We speculate that Wnt5a-containing sEVs do not modulate the PCP pathway at the mRNA level and instead engage PCP components at the protein level, though how, is subject to future studies.

Taken together, our data suggest that the axon elongation effects of the Wnt5a-CM are largely mediated by the Wnt5a associated with sEVs and that Wnt5a promotes axon elongation in a manner that requires endogenous Wnts and the components of PCP, Pk, and Vangl.

## 5. Conclusions

The current study elucidates a coordinated role of Wnt5a, endogenous Wnts, and Wnt–PCP components in axon outgrowth. We show that exogenous Wnt5a promotes axon elongation in mouse embryonic cortical neurons in a distinct timeframe compared with endogenous Wnts. Wnt5a-induced axon elongation requires the activity of the PCP components, Pk and Vangl. Moreover, we show that a substantial fraction of the Wnt5a present in the conditioned media is associated with sEVs and that Wnt5a-containing sEVs can promote axon outgrowth. Further studies are needed to identify the molecular components of sEVs that are responsible for the axon outgrowth effects and explore the underlying mechanistic details of Wnt5a-containing sEVs in axon outgrowth.

## Figures and Tables

**Table 1:** List of siRNAs.

Gene	Dharmacon Catalog #
*Prickle1*	MQ-042729-01-0002
*Prickle2*	MQ-056882-02-0002
*Vangl1*	MQ-057276-01-0002
*Vangl2*	MQ-059396-01-0002
*Porcupine*	MQ-049203-01-0002
*Wntless*	MQ-060922-01-0002

**Table 2:** List of antibodies.

Antibodies	Source	Identifier
Mouse anti- MAP2 (Clone AP20, 1:500)	Millipore (Oakville, ON, Canada)	MAB3418
Mouse anti-Tau-1 (Clone PC1C6, 1:300)	Millipore	MAB3420
Mouse anti-Tubulin β 3 (Clone Tuj1, 1:3000)	Biolegend (San Diego, CA, USA)	801202
Mouse anti-TSG101 (1:1500)	GeneTex (Irvine, CA, USA)	GTX70255
Mouse anti-Flotillin-1 (1:1500)	BD Transduction (Franklin Lakes, NJ, USA)	618021
Mouse anti-CD81 (B-11, 1:1500)	Santa Cruz (Dallas, TX, USA)	sc-166029
Goat anti-Wnt5a (1:1000)	R&D Systems	AF645
Rabbit anti-Actin (1:1000)	Sigma-Aldrich	A2066
Rabbit anti-Dvl2 (H-75, 1:1000)	Santa Cruz	sc-13974
Rabbit anti-Calnexin (1:2000)	[29]	N/A
Goat anti-Mouse Alexa Fluor^®^ 488 (1:1000)	Invitrogen	A11029
Goat anti-Mouse Alexa Fluor^TM^ 488 (1:500)	Thermo Fisher Scientific	A21121
Goat anti-Mouse Alexa Fluor^TM^ 568 (1:500)	Thermo Fisher Scientific	A21134
Goat anti-Mouse Alexa Fluor^®^ 594 (1:1000)	Invitrogen	A11032
Donkey anti-Goat IgG-HRP (1:5000)	Jackson ImmunoResearch (West Grove, PA, USA)	705-035-147
Donkey anti-Mouse IgG-HRP (1:5000)	Jackson ImmunoResearch	715-035-150
Goat anti-Rabbit IgG-HRP (1:5000)	Jackson ImmunoResearch	711-035-152

**Table 3:** List of qPCR primers.

Gene	Forward Primer	Reverse Primer
*Prickle1*	TCCCGAAACAAGGTCAGATTTA	TCTCTGGATCTGGCTGACT
*Prickle2*	CACTGCTTTGAGTCCCTGTATG	TCTGTAGCATGCCAGTGTTG
*Vangl1*	GCTGGCCTGAAAGTCTACAA	CGTGTTCGGCCTCTTCATAATA
*Vangl2*	ACTCGGGCTATTCCTACAAGT	TGATTTATCTCCACGACTCCCAT
*Wntless*	TGGGAAGCAGTCTAGCCTCC	GCAGCACAAGCCAAGGTGATA
*Porcupine*	GAGAAGGACCACCTGGAATG	ATAAGACATGGGCAGGTTCC
*Gapdh*	AGGTCGGTGTGAACGGATTTG	TGTAGACCATGTAGTTGAGGTCA

## Data Availability

The data presented in this study are available in the article and Supplementary Materials.

## Supplementary Materials

The following supporting information can be downloaded at: https://www.mdpi.com/article/10.3390/cells13151268/s1, Figure S1: Wnt5a promotes the growth of the longest neurite. Figure S2: Wnt5a promotes the elongation of the nascent axon. Figure S3: Wnt5a-containing sEVs have no effect on the expression of PCP components, Pk and Vangl.
