# Supplementary material for: Wnt5a Promotes Axon Elongation in Coordination with the Wnt–Planar Cell Polarity Pathway
Source: Cells. 2024 Jul 28;13(15):1268. doi: 10.3390/cells13151268 (PMC11312420; doi:10.3390/cells13151268)
Supplement: Supplementary file 1 [file cells-13-01268-s001.zip › cells-3068166-supplementary.pdf]

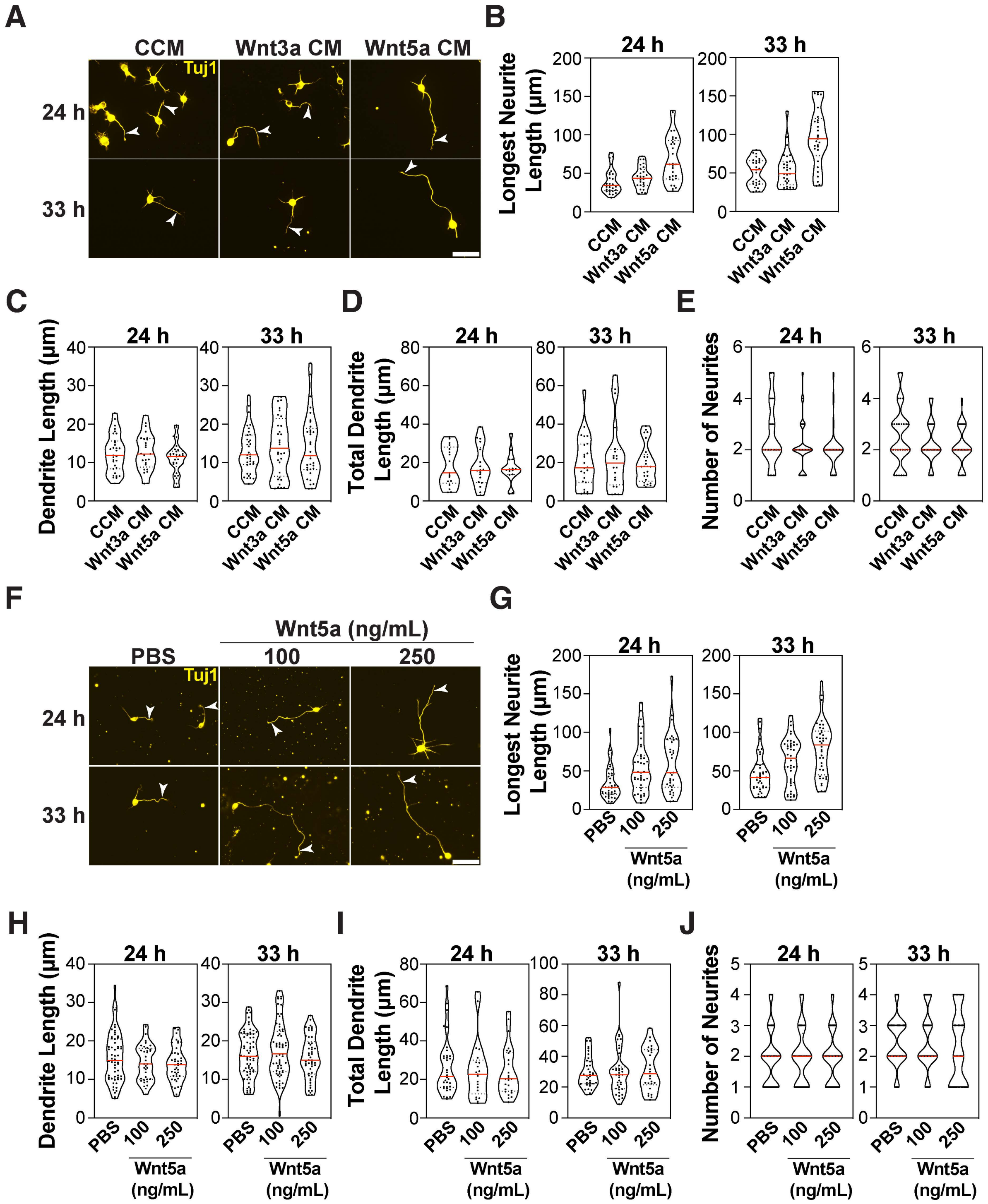

Figure S1. Wnt5a promotes the growth of the longest neurite.

(A-E, F-J) Cortical neurons are treated with the Wnt-CM (A-E) or commercially available recombinant Wnt5a (100 and 250 ng/mL) (F-J), 4 h after plating. Neurons were fixed at 24 and 33 h, and neuronal morphology was examined in Tuj1 stained neurons. Representative images are shown. Arrowheads mark the longest neurite. Scale bar, 40 μm (A, F). (B-E, G-J) The length of the longest neurite (prospective axon; B, G), individual dendrite lengths (C, H), total dendrite length (longest neurite excluded; D, I) and total number of neurites (E, J) were quantified from a minimum of 30 neurons per experiment and is shown as a representative violin plot with the median, marked in red, from 2 independent experiments.

Figure S1

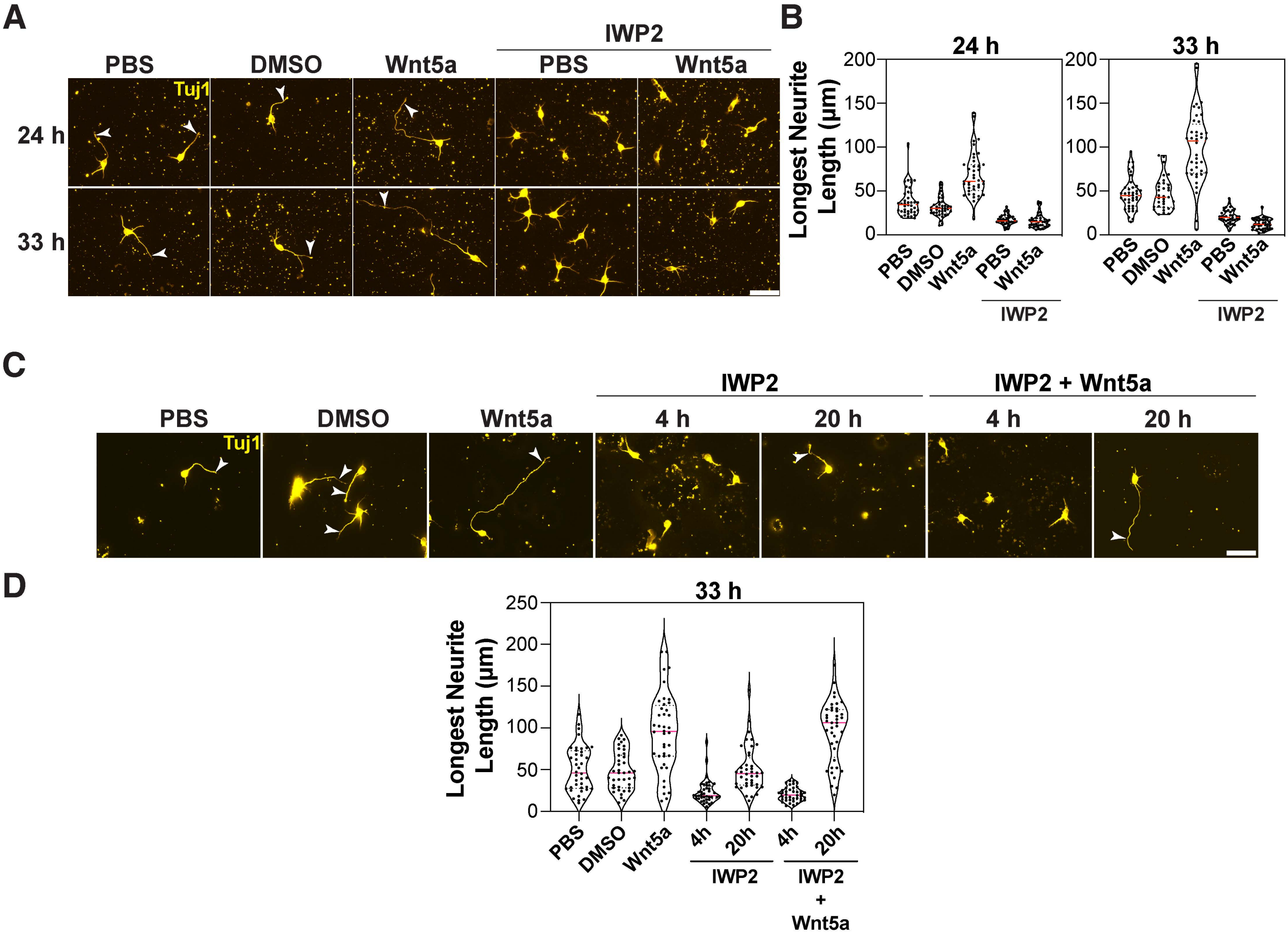

Figure S2. Wnt5a promotes the elongation of the nascent axon.

(A-D) Dissociated E15.5-16.5 mouse cortical neurons were treated with Wnt5a (100 ng/mL), 4 h after plating, in the absence or presence of IWP2 (10 µM), added 4 h (A, B) or 4 h and 20 h after plating (C, D). Neurons were fixed at 24 and 33 h (A, B) or 33 h (C, D) and neuronal morphology was examined in Tuj1 stained neurons. (A, C) Representative images are shown. Arrowheads mark the longest neurite. Scale bar, 40 µm. (B, D) The length of the longest neurite (prospective axon) is plotted as a representative violin plot from 2 independent experiments with median, marked in red, from a minimum of 30 neurons per experiment.

Figure S2

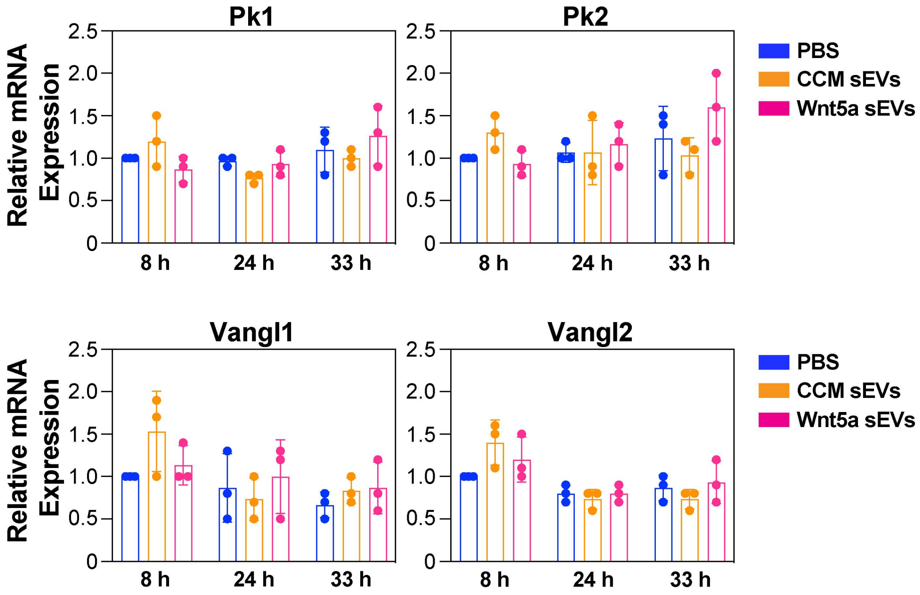

Figure S3. Wnt5a-containing sEVs have no effect on the expression of PCP components, Pk and Vangl.

Dissociated E15.5-16.5 mouse cortical neurons were treated with sEVs from control conditioned media (CCM) or Wnt5a-CM (all at 5 μg/mL), 4 h after plating. RNA was extracted at 8, 24 and 33 h and relative mRNA expression was determined by qPCR for the indicated genes. Data is plotted as mean ± SEM from 3 independent experiments.
